# Cluster detection of HIV infection for clients of female sex workers in selected districts of Tamil Nadu, South India

**DOI:** 10.1186/1471-2334-14-S3-O27

**Published:** 2014-05-27

**Authors:** Vasna Joshua, K Bhoopathi, Ramesh S Paranjape, Thilakavathi Subramanian, S Mehendale

**Affiliations:** 1 National Institute of Epidemiology, Ayapakkam, Chennai, India; 2 National AIDS Research Institute, Pune, India

## Background

The spread of the HIV epidemic is diverse. The risk behaviors of the HIV infected individuals are not distributed uniformly across the population, but tend to cluster in specific high risk groups. The objective of the study is to explore whether HIV infected individuals are geographically clustered using Kulldorff space time scan statistics.

## Methods

A large cross sectional Integrated Behavioral and Biological Assessment (IBBA) survey for clients of female sex workers (FSWs) in the three districts (Chennai, Madurai and Salem) of Tamil Nadu, South India was carried out by India AIDS initiative, the Avahan project. The survey collected information from 1217 clients of female sex workers between June and September 2009. The data set was geocoded using Google Earth, Kulldorff Space time Scan Statistics was used for detection of clusters among the clients of FSWs.

## Results

Kulldorff Space time Scan Statistics identified a most significant cluster at the location of latitude 13.123°N and longitude of 80.293°E with the spatial dependency of 8.39 km radius.

## Conclusions

The study shows initial evidence of geographical clustering of HIV cases. However this observation needs to be substantiated by undertaking similar studies in other states of India. Also, it is important to critically explore biological and behavioral reasoning for such an occurrence. This would also pave a way to build models incorporating clustering effect in the model and might help to design precise and more focused intervention approaches

